# Replacement of cysteine at position 46 in the first cysteine-rich repeat of the LDL receptor impairs apolipoprotein recognition

**DOI:** 10.1371/journal.pone.0204771

**Published:** 2018-10-17

**Authors:** A. Benito-Vicente, K. B. Uribe, H. Siddiqi, S. Jebari, U. Galicia-Garcia, A. Larrea-Sebal, A. Cenarro, M. Stef, H. Ostolaza, F. Civeira, L. Palacios, C. Martin

**Affiliations:** 1 Instituto Biofisika (UPV/EHU, CSIC) and Departamento de Bioquímica, Universidad del País Vasco, Bilbao, Spain; 2 Unidad de Lípidos and Laboratorio de Investigación Molecular, Hospital Universitario Miguel Servet, Instituto Aragonés de Ciencias de la Salud (IACS), Zaragoza, Spain; 3 Progenika Biopharma, a Grifols Company, Derio, Spain; University of Basque Country, SPAIN

## Abstract

**Background and aims:**

Pathogenic mutations in the Low Density Lipoprotein Receptor gene (*LDLR)* cause Familial Hypercholesterolemia (FH), one of the most common genetic disorders with a prevalence as high as 1 in 200 in some populations. FH is an autosomal dominant disorder of lipoprotein metabolism characterized by high blood cholesterol levels, deposits of cholesterol in peripheral tissues such as tendon xanthomas and accelerated atherosclerosis. To date, 2500 *LDLR* variants have been identified in the *LDLR* gene; however, only a minority of them have been experimentally characterized and proven to be pathogenic. Here we investigated the role of Cys46 located in the first repeat of the LDL receptor binding domain in recognition of apolipoproteins.

**Methods:**

Activity of the p.(Cys46Gly) *LDLR* variant was assessed by immunoblotting and flow cytometry in CHO-*ldlA7* expressing the receptor variant. Affinity of p.(Cys46Gly) for LDL and VLDL was determined by solid-phase immunoassays and *in silico* analysis was used to predict mutation effects.

**Results and conclusion:**

Functional characterization of p.(Cys46Gly) *LDLR* variant showed impaired LDL and VLDL binding and uptake activity. Consistent with this, solid-phase immunoassays showed the p.(Cys46Gly) *LDLR* variant has decreased binding affinity for apolipoproteins. These results indicate the important role of Cys46 in LDL receptor activity and highlight the role of LR1 in LDLr activity modulation. This study reinforces the significance of *in vitro* functional characterization of LDL receptor activity in developing an accurate approach to FH genetic diagnosis. This is of particular importance because it enables clinicians to tailor personalized treatments for patients’ mutation profile.

## Introduction

Familial Hypercholesterolemia is an autosomal dominant disorder causing premature coronary heart disease (CHD) [1] characterized by high blood cholesterol levels, deposits of cholesterol in peripheral tissues such as tendon xanthomas and accelerated atherosclerosis [2]. With a heterozygous frequency prevalence as high as 1 in 200 in some populations [3, 4], FH is frequently underdiagnosed [4, 5]. Most often, FH is caused by mutations in the Low Density Lipoprotein Receptor gene (*LDLR*; MIM# 606945), which removes both Low-Density Lipoproteins (LDL) and Very Low Density Lipoproteins (VLDL) from the bloodstream [6]. Because FH patients usually respond well to medication, early diagnosis of FH can significantly reduce the risk of CHD [7]. Genetic screening for *LDLR* pathogenic variants is a cost-effective strategy to enable such early diagnoses [8]. As of August 31, 2018, a total of 2500 *LDLR* variants have been identified in the ClinVar Database (https://clinvarminer.genetics.utah.edu). Since this database includes pathogenic variants, non-pathogenic variants and variants with conflicting interpretation, distinguishing pathogenic variants from non-pathogenic ones is a long-standing challenge in the field [9].

The ability of LDL receptor (LDLr) to bind both LDL and VLDL is mediated by interactions between the LDLr Ligand Binding Domain (LBD) and the apolipoprotein components of VLDL and LDL [10]. While LDL is composed of a single copy of apolipoprotein (apo) B, VLDL are heterogeneous particles that contains one copy of apo B and a variable number of copies of the smaller apo E and/or apo CIII [11, 12], [13].

The LBD consists of seven cysteine-rich ligand-binding repeats (LRs) of approximately 40 residues each [14]. Each LR contains a single structural calcium ion [15] as well as six cysteines that interact via Cys(I)-Cys(III), Cys(II)-Cys(V) and Cys(IV)-Cys(VI) disulfide bonds [16]. In each LR, the six cysteines surround a highly conserved negatively charged sequence of Ser-Asp-Glu near the LR C-terminus [17]. This negatively charged sequence interacts with positively charged residues on apo B and apo E to enable lipoprotein binding [18]. LR4 and LR5 play a mayor role in LDL particle recognition [10, 17], whereas deletion of individual repeats LR2–LR7 reduces LDL binding and, deletion of LR1 has little effect on lipoprotein binding [10, 17]. In addition, binding of VLDL or its remnants (β-VLDL) to LDL receptor involves simultaneous binding of apo E copies to the LR5 along with additional repeats of the LDLr[19]. Weak interactions between apo E and LR3, LR4 and LR5 have also been described [20, 21].

The LR1 comprises 40 amino acids, which account for the 4.7% of the protein, whereas the percentage of LR1 different missense variants found in the *LDLR* represent the 2.57% of the missense described variants (ClinVar database). This percentage is in concordance with the fact that the LR1 domain seems to be implicated to a lesser extent than LR2-LR7 in LDL and VLDL binding. It is noteworthy that the cysteine missense variants represent the 42.3% of the amino acid changes in LR1 reported in the ClinVar in relation to Hypercholesterolemia (actualized August 31, 2018). Among them, mutations at Cys46, the fourth cysteine of the LR1 domain, have been described as pathogenic when the Cys is replaced by a Ser [22], while replacement by a Gly or Tyr has not yet been functionally characterized [23, 24], unfortunately none of them has been functionally characterized.

In this study, in order to gain insight into the relevance of LR1 in LDL and VLDL binding activity, we addressed the activity and affinity of LDLr with the substitution p.(Cys46Gly) to LDL and VLDL. The selection of the p.(Cys46Gly) LDLr variant was based on three criteria: our interest in studying mutations in LR1 region that could have a pathogenic effect (despite this region has been considered no essential for LDLr activity[10, 17], previous documentation of this variant in FH patients, and, obviously, to study a variant whose effect has not been characterized before. Our results show that substitution of Cys46 by a Gly leads to a loss of LDLr binding affinity for LDL leading to loss of binding and uptake, as determined by solid-phase immunoassays and flow cytometry, respectively. In addition VLDL uptake results also impaired as a consequence of p.(Cys46Gly) substitution. Our results highlight the role of LR1 in modulating the activity of LDLr and help in a better understanding of the potential mechanisms of pathogenicity related to mutations in the LR1.

## Materials and methods

### *In silico* predictions of p.(Cys46Gly) LDLr variant activity

This variant has been included in the ClinVar database (https://clinvarminer.genetics.utah.edu) by 3 submitters (accession code: RCV000238380) and has also been found in 3 FH index cases by Progenika Biopharma (Derio, Spain). The *in silico* analysis of the selected variant is compiled in Table 1.

**Table 1:** Description of p.(Cys46Gly) LDLr variant, conservation and *in silico* predictions.

**Genetic name**	**HGVS Nomenclature**	**Conservation nt**	**Conservation AA**	
c.136T>G	p.(Cys46Gly)	1.00	1.00	
**Pathogenicity prediction**	**SIFT**	**Align GVGD**	**POLYPHEN-2**	**Mutation taster 2**
	Deleterious (score 0)	Pathogenic (C0)	Probably damaging (1)	Disease causing (1.0)

### Site-directed mutagenesis

Plasmid carrying the p.(Cys46Gly) *LDLR* variant was constructed by Innoprot (Derio, Spain). Briefly, variants were introduced into the human *LDLR* cDNA (NM_000527.4), using the mammalian expression vector pcDNA3 under control of a SV40 promoter by oligonucleotide site-directed mutagenesis using the QuickChange Lightning mutagenesis kit (Agilent) according to the manufacturer’s instructions. The oligonucleotides used to generate the plasmid carrying p.(Cys46Gly) *LDLR* variant were synthesized *in vitro* and subcloned using the restriction enzymes SacII and EcoRI. The presence of the desired nucleotide alteration was confirmed by PCR and restriction enzyme digestion of the appropriate fragments. The integrity of the remaining *LDLR* cDNA sequence of the construct was verified by direct sequence analysis.

### LDL receptor-ectodomain production and purification

The LDL receptor ectodomain (1–789 amino acids) plus c-myc and His tags in both wild type (wt) and p.(Cys46Gly) LDLr variant were purified from cells transfected with the pcDNA3.1-EC-LDLR-His plasmid, kindly provided by Prof. Leren [25] and pcDNA3.1-EC-Cys46GlyLDLR-His plasmid, respectively. Briefly, HEK293 cells were transfected with the plasmid by calcium phosphate method for 24–48 h and selected by geneticin (G-418 sulphate, Gibco, Invitrogen). The LDLr ectodomain was affinity purified using one-step nickel affinity chromatography as described before [26]. Cys46Gly variant was introduced by oligonucleotide site-directed mutagenesis using QuickChange Lightning mutagenesis kit (Agilent) according to manufacturer’s instructions and using 5’-TAC AAG TGG GTC GGC GAT GGC AGC GC-3’ and 5’-GCG CTG CCA TC GCC GAC CCA CTT GTA-3’ forward and reverse primers, respectively.

### Cell culture and transfection

*LDLR*-deficient CHO-*ldl*A7 cells (provided by Dr. Monty Krieger, Massachusetts Institute of Technology, Cambridge, MA) were cultured in Ham’s F-12 medium containing 10% fetal bovine serum (FBS), 2 mM L-glutamine (Invitrogen), 100 units/mL penicillin, and 100 μg/mL streptomycin (Invitrogen). Transfections were carried out using Lipofectamine LTX and Plus Reagent (Invitrogen) in 6- or 24-well culture plates according to the manufacturer’s instructions. The experiments were carried out 48 h post-transfection.

### Western blot analysis

Cell lysates were prepared, using an ice-cold buffer containing 50 mM Tris–HCl, pH 7.5, 125 mM NaCl, 1% Nonidet P-40, 5.3 mM NaF, 1.5 mM NaP, 1 mM orthovanadate, 1 mg/ml protease inhibitor cocktail (Roche), and 0.25 mg/ml Pefabloc, 4-(2-aminoethyl)-benzenesulfonyl fluoride hydrochloride (AEBSF; Roche). Cells were sonicated for 10 pulses at 10 kHz on ice, rotated at 4°C for an hour and centrifuged at 12,000g during 15 minutes to remove insoluble material. Proteins were fractionated by electrophoresis on non-reducing 8.5% SDS-PAGE for semi-quantitative immunoblotting. The following antibodies were added: rabbit polyclonal anti-LDLr antibody (1:500) (Progen Biotechnik GmbH, Heidelberg, Germany), anti-Glyceraldehyde 3-phosphate dehydrogenase (GAPDH) antibody (1:1000) (Nordic Biosite, Täby, Sweden) and horseradish peroxidase-conjugated anti-rabbit antibody (GE Healthcare, Little Chalfont, UK). The primary antibodies were incubated overnight at 4°C while the secondary antibody incubation was performed at room temperature for an hour. Signals were developed using SuperSignal West Dura Extended Substrate (Pierce Biotechnology, Rockford, IL, USA) in a ChemiDoc XRS (Bio-Rad, Hercules, CA, USA). NIH ImageJ software (http://rsbweb.nih.gov/ij/) was used for band intensity quantification, levels of protein were corrected to GAPDH loading control band intensities.

### Lipoprotein isolation

Plasma used for lipoprotein purification was collected from healthy individuals blood after 30 min centrifugation, at 12,000 x g at 4°C. The samples were then adjusted with KBr to d ¼ 1.225 kg/L and a second PBS buffer phase was added in the top. The ultracentrifugation was carried out in a TST SW-28ti (Beckman) at 27,000 rpm for 22 h at 4°C in a Beckman Optima L-90K. The white upper band corresponding to VLDL and the intermediate orange band corresponding to LDL were collected and stored at 4°C. Isolated lipoproteins were used within 2–3 days after purification.

### Solid-phase immunoassay for LDL-LDL receptor ectodomain binding

LDLr ectodomain fragments diluted in working buffer (10 mM Tris-HCl, pH 7.5, 50 mM NaCl, 2 mM CaCl_2_) were coated at a fixed concentration onto 96-well microtiter plates by incubation overnight at 4°C. Plates were then blocked and incubated with a serial dilution of LDL diluted in working buffer during 2 hours at room temperature, and then washed thoroughly with working buffer supplemented with 0.1% (w/v) Tween 20 (Sigma-Aldrich, MO, USA). For ligand detection, the antibodies (goat polyclonal anti-apoB, abcam,UK; and peroxidase-conjugated mouse anti-goat, ThermoScientific, USA) were diluted in working buffer supplemented with 5% (w/v) BSA, applied directly to the plate and incubated for 1 hour at room temperature, with an extensive washing between both incubations. After a final wash, antibody binding was determined using 50 μL per well of 2,2´-Azino-bis (3-ethylbenzothiazoline-6-sulfonic acid) substrate solution (Sigma-Aldrich, MO, USA) and measuring colour change at 405 nm. The time course for colour development was essentially lineal and measurements were taken 30–60 min after the addition of substrate. For data processing, all absorbance values were corrected for unspecific binding, relativized to maximum and EC_50_ values were extracted from curves after fitting the data to 5-parameter logistic (5-PL) equation (SigmaPlot 13.0, Systat Software Inc., CA, USA).

### Lipoprotein labelling

LDL and VLDL were labelled with FITC as previously described [27]. Briefly, lipoproteins were loaded in a 0.1 M NaHCO_3_ (pH 9.0) pre-equilibrated Sephadex G-25 column and incubated with 10 μl/mL FITC (2 mg/mL in dimethyl sulfoxide) per lipoprotein millilitre (1mg/mL). The solution was mixed during 2 h at room temperature and then a Sephadex G-25 column was used to remove free FITC. Lipoprotein quantification was determined by Pierce BCA protein assay.

### LDLr expression determined by FACS

To determine LDLr cell surface expression by FACS the following antibodies were used: mouse primary antibody anti-LDLr (1:100; 2.5 mg/L; Progen Biotechnik GmbH), rabbit primary anti-LDLr polyclonal antibody (1:100; Cayman Chemical), secondary antibody Alexa Fluor 488-conjugated goat anti-mouse IgG (1:100; Molecular Probes) and Alexa Fluor 488-conjugated goat anti-rabbit IgG (1:100; Molecular Probes). The immunostaining was performed as previously described [28]. Briefly, cells were incubated for 1 hour at room temperature with the primary antibody after consecutive fixing and blocking steps. Cells were finally washed 3 times in PBS-1%BSA and incubated for 1 hour at room temperature with the secondary antibody. For each sample, fluorescence of 10,000 events was acquired for data analysis. All measurements were performed at least in triplicate.

### Quantification of LDLr activity by flow cytometry

Cells were seeded in 24-well plates, at 10^6^ cells/well and transfected as previously described when optimal concentration was reached. 48 hours after transfection, cells were incubated with 20 μg/mL FITC-LDL or FITC-VLDL within 4 hours at 37°C or at 4°C to determine LDLr activity and its binding to the different lipoproteins respectively. After incubation, cells were washed three times with PBS-1%BSA, fixed for 10 minutes in 4% paraformaldehyde and washed again to remove the remaining fixative.

FITC-LDL or FITC-VLDL uptake was determined by adding Trypan blue solution 0.2% final concentration (Sigma-Aldrich, Steinheim, Germany) to the samples thus quenching the extracellular fluorescence of the non-internalized lipoproteins. Fluorescence acquisition was performed in a FACScalibur Flow cytometer as previously described [29]. For each determination, at least 10,000 events were acquired and analysed with the CellQuest software system (Beckton and Dickinson).

### Statistical analysis

All measurements were performed at least 3 times, with at least 3 independent determinations for each measurement. Results shown in Table 2 and figures represent mean ± standard error of mean (S.D). A two-tailed Student’s t-test was used to determine levels of significance.. p-values lesser than or equal to 0.05 were considered significant.

**Table 2:** LDL affinity for wild type and p.(Cys46Gly) LDLr variant.

**LDL-LDLr ectodomain dissociation constant (KD) pH 7.4**		
	Kd (nM)	S.D
wt	0.58	0.11
p.(Cys46Gly)	15.79	2.05
**LDL-LDLr ectodomain dissociation constant (KD) pH 5.2**		
	Kd (nM)	S.D
wt	7.01	2.02
p.(Cys46Gly)	4.15	0.86

## Results

### *In silico* functional predictions of p.(Cys46Gly) LDLr variant

Four software programs were used to predict pathogenicity of p.(Cys46Gly) LDLr variant. As shown in Table 1. p.(Cys46Gly) variant was predicted as pathogenic by SIFT, GVGD and Mutation taster2 programs and as probably damaging by Polyphen-2. Because the prediction algorithms are mainly based on amino acid conservation analysis and Cys46 is highly conserved, the obtained predictions were the expected ones. To confirm these predictions, *in vitro* functional validation of LDLr variant was next assayed.

### Expression of LDLr variants in CHO-*ldl*A7 cells

**Fig 1:**
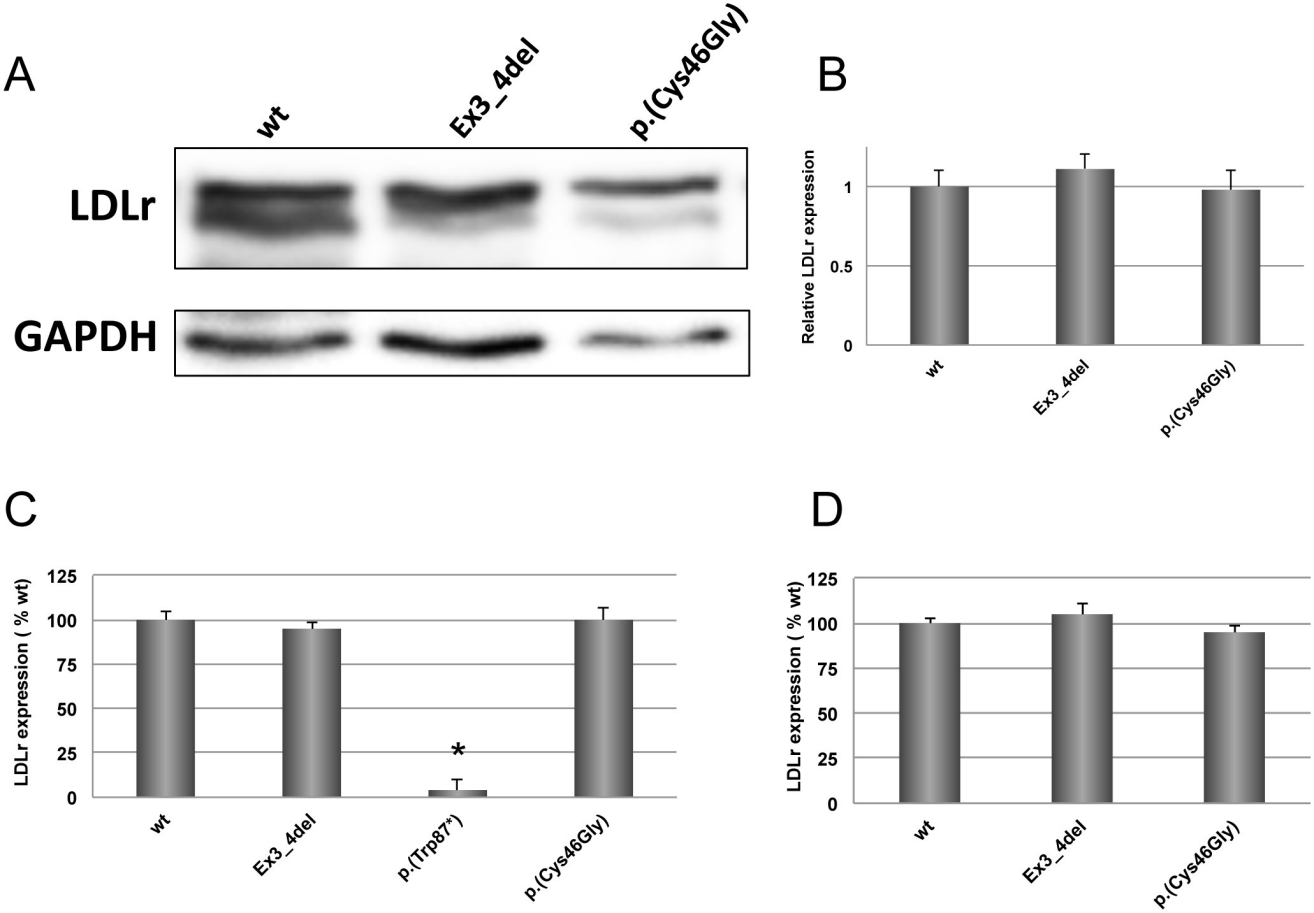
Expression of wt LDLr and p.(Cys46Gly) LDLr variant in CHO-*ldlA7* transfected cells. Expression of LDLr was assessed 48 h post-transfection with the corresponding plasmids by **A)** Western blot, **B)** mature LDLr protein relative to GAPDH. LDLr expression of p.(Cys46Gly) LDLr variant determined by FACS with **C)** IgG-C7 antibody that recognizes the N-terminal ligand binding repeat of the LDLr and **D)** a polyclonal anti- LDLr antibody. A representative blot is shown in panel A. The values in C and D represent the mean of triplicate determinations (n = 3); error bars represent ±SD. *P < 0.001 compared to wt using a Student’s t-test.

Expression of p.(Cys46Gly) LDLr variant was analysed by Western blot in CHO-*ldl*A7 transfected cells as described in *Materials and Methods*. LDLr signal (Fig 1A, upper panel) was normalized with GAPDH levels (Fig 1A, lower panel) and LDLr expression levels were quantified by densitometry (Fig 1B). Accordingly, p.(Cys46Gly) LDLr variant is expressed at similar levels as wt 48 h after transfection. Ex3_4del LDLr variant that produces a binding-defective LDLr was used as internal controls [10]. These results were also corroborated by determining LDLr expression by flow cytometry. Using both a IgG-C7 antibody recognizing the N-terminal ligand binding repeat of the LDLr and a polyclonal anti-LDLr antibody, we found that LDLr expression of p.(Cys45Gly) was similar to wt (Fig 1C and 1D, respectively).

### Affinity of wt LDLr and p.(Cys46Gly) LDLr variant for LDL

Next, we tested binding affinities of wt and p.(Cys46Gly) LDLr for LDL using a solid-phase binding immunoassay. The EC_50_ for wt LDLr to LDL was calculated to be 0.58 ± 0.11 nM, very similar to previously reported values[30, 31]. Consistently with the results obtained by flow cytometry, the p.(Cys46Gly) LDLr variant showed a significant lower affinity for LDL (15.79 ± 2.05 nM, p<0.01) (Table 2). Binding affinities of LDL-LDLr and LDL- p.(Cys46Gly) LDLr variant were also determined at pH 5.2, as expected wt LDLr showed a lower affinity for LDL at acidic pH with a Kd = 7.01 ± 2.02 nM. Interestingly, p.(Cys46Gly) LDLr showed a higher affinity to LDL (4.15 ± 0.86 nM, p<0.01) at pH 5.2.

### Analysis of LDLr activity in CHO-*ldl*A7 cells

**Fig 2:**
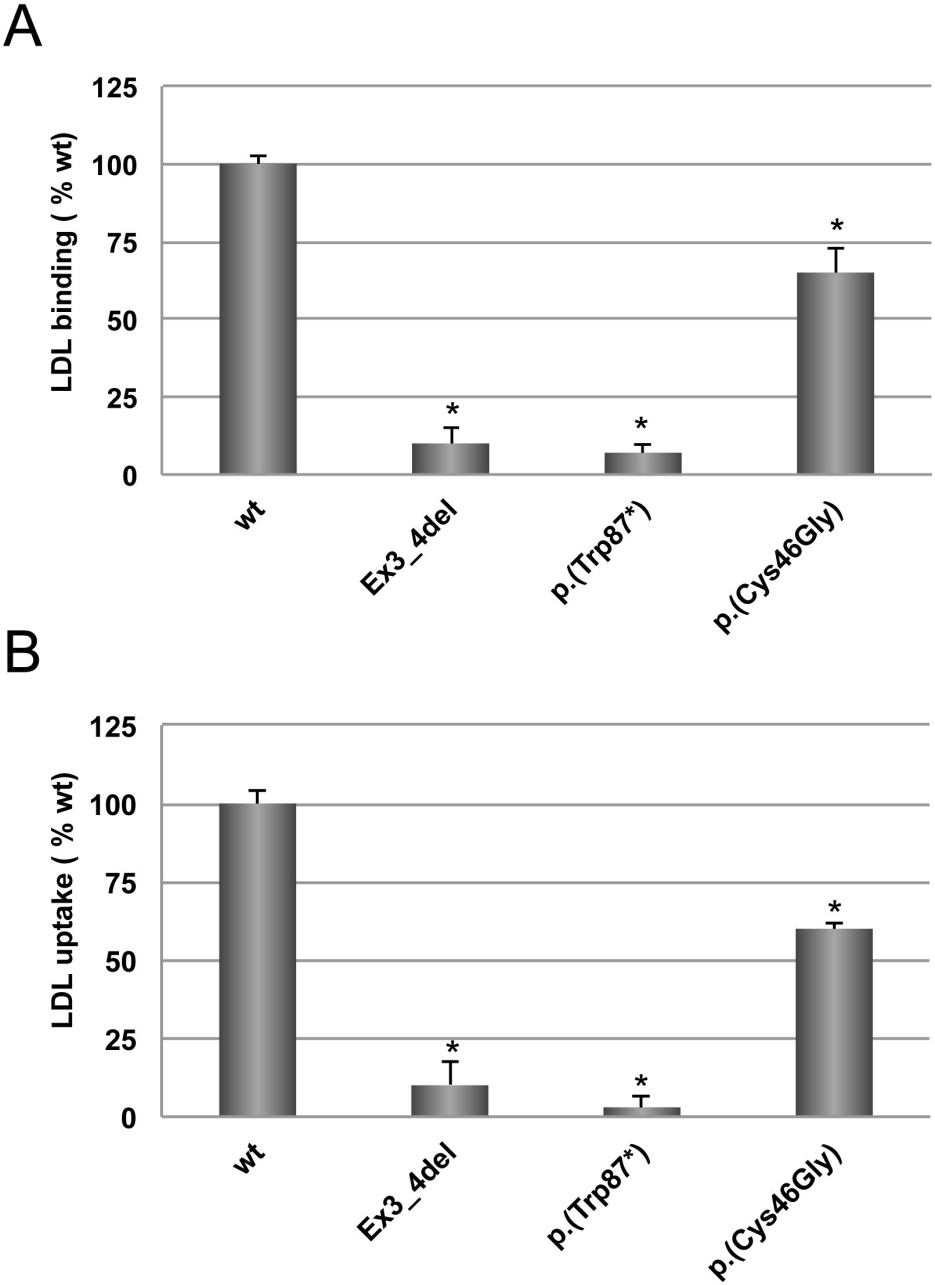
LDLr activity of wt and p.(Cys46Gly) LDLr variants. **A)** LDL- LDLr binding and **B)** FITC-LDL uptake activity. Assays were performed as described in Materials and Methods. Data show the mean of three independent experiments; error bars represent ±SD. *P < 0.001 compared to wt using a Student’s t-test.

CHO-*ldl*A7 cells expressing wt or p.(Cys46Gly) LDLr variant were assayed for LDL binding and uptake by flow cytometry. Two internal controls were used: p.(Trp87*) (a null allele mutant), and Ex3_4del *LDLR* variant that produces a binding-defective LDL receptor [10]. As shown in Fig 2A, LDL- LDLr binding activity of p.(Cys46Gly) variant resulted in 35% lower compared to wt LDLr (100 ± 3 vs. 65 ± 8, p<0.01) (Fig 2A). As shown in Fig 2B and in agreement with LDLr binding results, LDL internalisation in cells expressing p.(Cys46Gly) variant was significantly diminished (≈ 40% reduction, 100 ± 4 vs. 60 ± 2, p<0.01) when compared to wt.

### VLDL (E3/E3) uptake in CHO-*ldl*A7 cells

**Fig 3:**
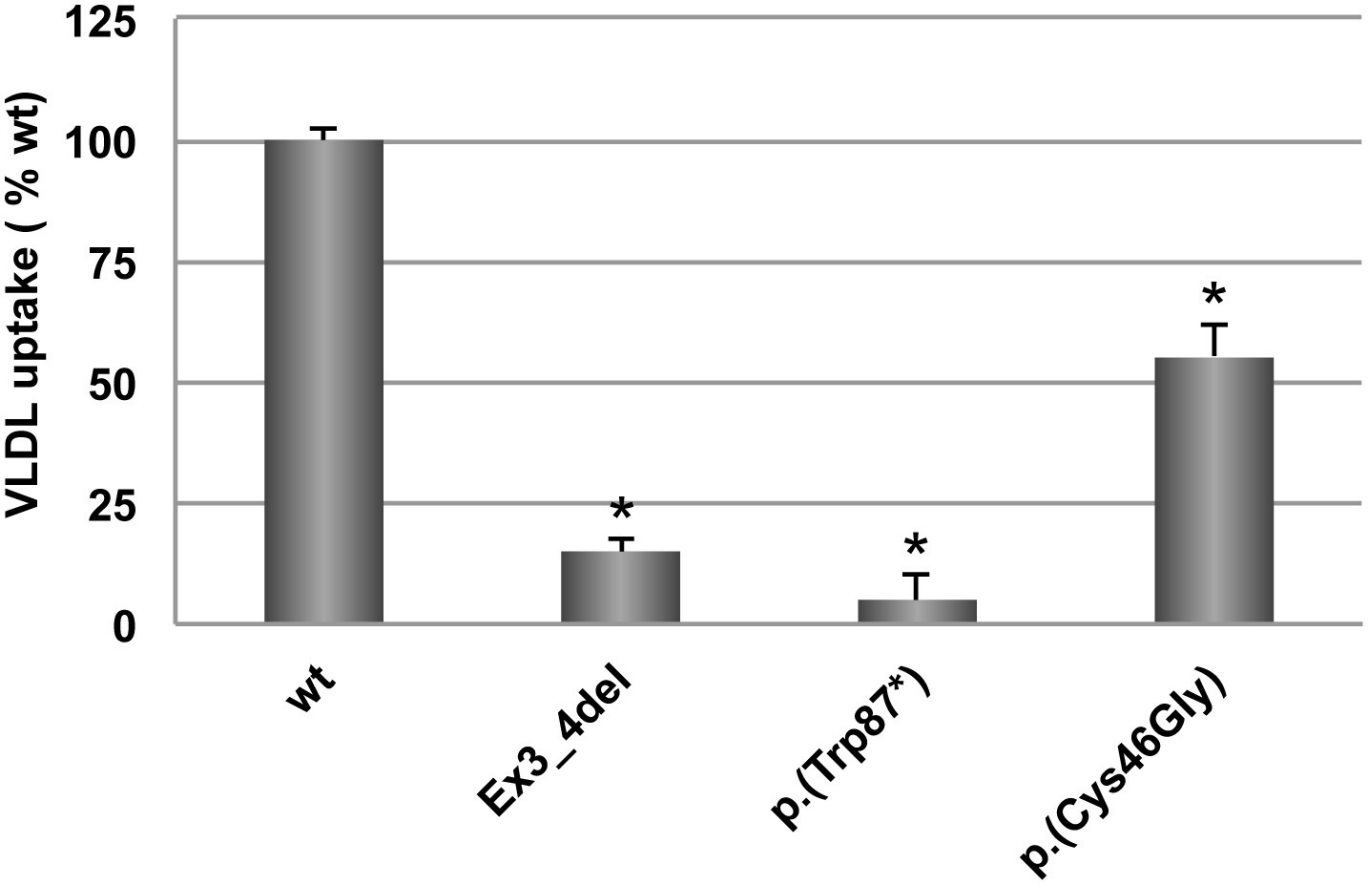
Analysis of VLDL uptake in wt and p.(Cys46Gly) LDLr variant. VLDL uptake activity. Assays were performed as described in Materials and Methods using FITC-labelled VLDL. Data show the mean of three independent experiments; error bars represent ±SD. *P < 0.001 compared to wt using a Student’s t-test.

CHO-*ldl*A7 cells transfected with wt or p.(Cys46Gly) LDLr variant were used to determine if the amino acid substitution impairs apoE3 VLDL uptake activity. In this assay, Ex3_4del mutant was also used as an internal control because it lacks the LR4 module, which, together with LR5, is required for VLDL binding. As shown in Fig 3, VLDL uptake in p.(Cys46Gly) LDLr variant was significantly decreased (≈ 45% reduction, 100 ± 2 vs. 55 ± 7, p<0.01) when compared to wt, (Fig 3).

## Discussion

This study has been focused on the p.(Cys46Gly) LDLr variant, previously associated with FH but not functionally characterized [23]. Despite being classified as pathogenic in the ClinVar database (https://clinvarminer.genetics.utah.edu) by two of the 3 submitters, all prior classification criteria was based only on predictions. In order to gain insight into the role of LR1 in LDLr activity we experimentally determined *in vitro* the functionality of p.(Cys46Gly) LDLr variant. The data obtained by solid-phase immunoassays and FACS show that replacement of Cys46 by a glycine diminishes the affinity for both LDL and VLDL thus negatively modulating LDLr activity, emphasising the role of LR1 on LDLr activity.

The binding domain of LDLr to lipoproteins is organized into seven cysteine rich domains called LR1-LR7 [32], each of which is composed of about 40 amino acids and stabilized by three disulphide bridges [16]. The coordination of each LR with one Ca^2+^ ion facilitates LR folding in a way that makes the mature LR capable of lipoprotein binding [33].

In particular, the role of LR1 in binding lipoproteins has been highly controversial. Deletion of the whole LR1 from LDLr affects neither LDL nor VLDL binding [10, 17]. Consistent with this, truncated proteins containing only LR1 are unable to bind any lipoprotein [34]. These findings initially led to the erroneous conclusion that LR1 is not involved in lipoprotein binding [34]. Later studies, however, found that lipoprotein binding is more likely coordinated by a combination of multiple LRs including LR1 [34]. While deletion of the whole LR1 does not alter lipoprotein binding, missense mutations in LR1 can significantly alter lipoprotein binding[34]. The number of variants located at LR1 is lower than the number found at LR2-LR7, 26 variants compared to 50–72, according to ClinVar database. Among these 26 missense variants, 11 are classified as pathogenic by at least 1 submitter but only the ones impairing a Cys have been really demonstrated to be pathogenic by a functional study, the rest of them have not enough data to support their classification as pathogenic. This finding at least suggests that substitutions at the cysteine residues of the LR1 can be responsible for reduced LDLr activity leading concomitantly to FH.

Specifically, in the case of p.(Cys46Gly) LDLr variant, substitution of a cysteine by glycine can disturb the structure of LR1, since loss of the CysIV-CysVI disulphide bridge can lead to several disulphide-bonded isomers. Because the cysteine amino acid plays a key role in maintaining integrity of the binding subunits, replacement by other amino acid would allow neither proper folding of the LR nor calcium coordination[35, 36] as previously demonstrated in cysteine substitutions in LDLr located in the LBD [35, 37]. Our findings here, however, show that the IgG-C7 antibody, which recognizes a structural epitope at the N-terminus of LR1, is still able to bind the receptor. This would indicate that this epitope in LR1 is still intact, and for this reason, we suggest that the substitution of Cys at position 46 by Gly does not completely destabilize the LR1 structure. Moreover, expression of p.(Cys46Gly) LDLr variant is similar to that of wt LDLr indicating a minimal contribution of other disulphide-bonded isomers that probably are retained in the endolasmic reticulum and ultimately degraded [38].

Taking together that the first binding repeat is not essential for LDL recognition [39] and that the integrity of the first repeat is at least not dramatically affected, it could be expected that the p.(Cys46Gly) LDLr variant would not affect the activity of LDLr. Nevertheless, the diminished LDLr activity demonstrated by our experimental data suggests that the cysteine at position 46 itself plays an active role in receptor activity. Several amino acid substitutions by glycines lead to an increased elasticity and mobility of the proteins because of the short side chain of the glycines. These changes allow positional variations of the residues within the protein, even without a significant change in R1 folding. This in turn, could slightly modify the structure of the contiguous binding repeats and contribute to a loss of LDL binding activity [10].

In summary, we found that the p.(Cys46Gly) LDLr variant has a diminished LDL and VLDL binding activity, as assessed by solid-phase immunoassay and FACS using CHO-*ldl*A7 cells. Because the used experimental models mimic the presence of the variant at homozygous status, the 40–45% of activity reduction in the p.(Cys46Gly) LDLr variant indicates the variant is mildly pathogenic. This is especially relevant because *LDLR* mutations with similar effects in heterozygous state could go unnoticed due to masking by the wildtype *LDLR* allele. In addition, the different severity of the mutations can explain the high variability found in the FH phenotype. Our findings here reinforce that *in vitro* functional characterization of LDLr variants is a gold standard in genetic diagnosis of Familial Hypercholesterolemia, particularly when the LDLr activity is only partially reduced, as is the case of the p.(Cys46Gly) variant.
